# The Impact of *Lactobacillus reuteri* on Oral and Systemic Health: A Comprehensive Review of Recent Research

**DOI:** 10.3390/microorganisms13010045

**Published:** 2024-12-30

**Authors:** Zihui Liu, Qing Cao, Wenqing Wang, Bowen Wang, Yilun Yang, Cory J. Xian, Tiejun Li, Yuankun Zhai

**Affiliations:** 1 School of Stomatology, Henan University, Kaifeng 475004, China; 15138959362@163.com (Z.L.); 19937823302@163.com (Q.C.); 15937820049@163.com (W.W.); wangbw512@163.com (B.W.); yyl13450945213@163.com (Y.Y.); litiejun22@vip.sina.com (T.L.); 2 Kaifeng Key Laboratory of Periodontal Tissue Engineering, Kaifeng 475000, China; 3 UniSA Clinical and Health Sciences, University of South Australia, Adelaide, SA 5001, Australia; cory.xian@unisa.edu.au; 4 Department of Oral Pathology, Peking University School and Hospital of Stomatology, Beijing 100081, China

**Keywords:** *Lactobacillus reuteri*, microbiome, periodontal diseases, oral mucosal diseases, dental caries, gastrointestinal diseases

## Abstract

Oral diseases, particularly dental caries and periodontal disease, pose significant global health challenges. The imbalance of the oral microbiota plays a key role in the occurrence of these diseases, prompting researchers to seek new strategies to restore oral ecological balance. *Lactobacillus reuteri* is a Gram-positive rod-shaped bacterium that exists in various body parts of humans, including the gastrointestinal tract, urinary tract, skin, and so on. This species has a potentially positive impact on oral health and plays an important role in maintaining systemic health. Recent studies have explored the application of *Lactobacillus reuteri* in the prevention and treatment of oral diseases, and its impact on systemic health has also been preliminarily revealed. The current review summarizes the role of *Lactobacillus reuteri* in oral health and systemic health and outlines its potential applications in the future. *Lactobacillus reuteri* has shown promising prospects in treating non-communicable biofilm-dependent oral diseases, but its mechanism of action and efficacy still need further research. In addition, *Lactobacillus reuteri* has also displayed some potential benefits in promoting overall health. Future research should focus on revealing the specific pathways of action of *Lactobacillus reuteri*, screening for the most beneficial strains, determining the most effective drug delivery strategies, developing oral and systemic health products based on *Lactobacillus reuteri*, and ensuring their safety in clinical applications.

## 1. Introduction

**Figure 1:**
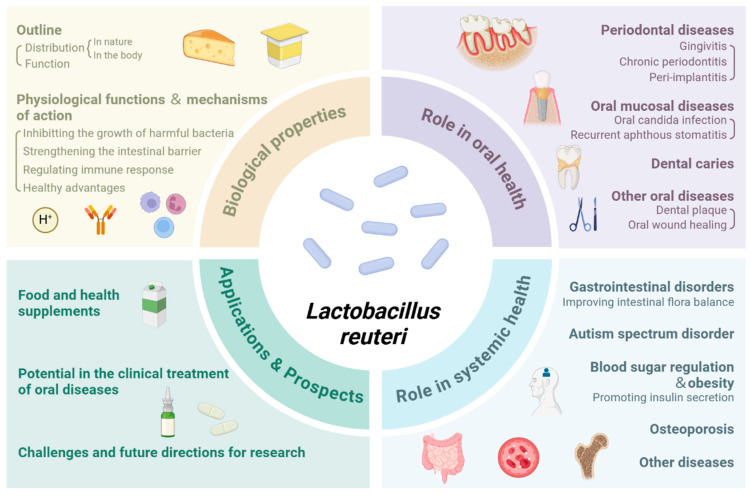
An overview of *Lactobacillus reuteri* in oral health, its biological characterization, roles in oral and systemic health, and prospects in practical applications. Created in https://BioRender.com (accessed on 25 November 2024).

Microbiota and oral health are intimately linked as they both influence and depend on each other [1]. In addition to its potentially advantageous roles, the imbalance of the oral microbiota can result in several oral illnesses and even endanger systemic health. With in-depth microbiome studies, regulating oral microbiota by increasing probiotics and reducing pathogenic bacteria has become one of the research directions for improving oral health [2,3,4,5]. Among them, *Lactobacillus reuteri* has become a study hotspot in recent years, which has considerable probiotic capabilities and presents in nearly all vertebrate and mammalian species by the ability to form symbiotic connections with other bacteria [6,7,8]. This article reviews the biological traits of *Lactobacillus reuteri*, its function in oral and systemic health, mechanisms of action, and potential and prospects in practical applications (Figure 1). 

## 2. Biological Characterization of *Lactobacillus reuteri*

### 2.1. Outline

*Lactobacillus reuteri* has caught much attention for its functional and unique role in a variety of diseases. *Lactobacillus reuteri* is present in plants, silage, and fermented foods, including cheese, yogurt, pickles, olives, and sausages [9]. It is one of the few *Lactobacilli* specifically adapted to survive in the gastrointestinal tract (which can colonize the oral cavity and gastrointestinal tract), urinary tract, and skin in the human body [10,11]. It is found throughout the oral cavity, including saliva, palate, tongue, tooth surfaces, gums, and periodontal tissues. It can establish a robust and densely packed biofilm with other bacteria on mucosal surfaces, obstructing the adherence of foreign pathogens. Moreover, it impacts oral health and systemic health by modulating oral microbiota, regulating immunological function, and producing bioactive compounds. Its colonization can be influenced by many factors, including genetic predisposition, age, gender, personal hygiene practices, dietary intake, and the form and dosage of probiotic supplementation [12,13].

According to incomplete statistics, there are dozens of strains of *Lactobacillus reuteri.* Among them, DSM 17938 and ATCC 55730 (also known as SD2112) are often used in the study of gastrointestinal disorders or as food additives to improve the gastrointestinal health of human beings [14], and DSM 17938, ATCC PTA 5289, ATCC 55730, KCTC3594, and KCTC3678 are also found in studies of oral diseases [15,16,17,18,19].

### 2.2. Physiological Functions and Action Mechanisms of Lactobacillus reuteri

**Figure 2:**
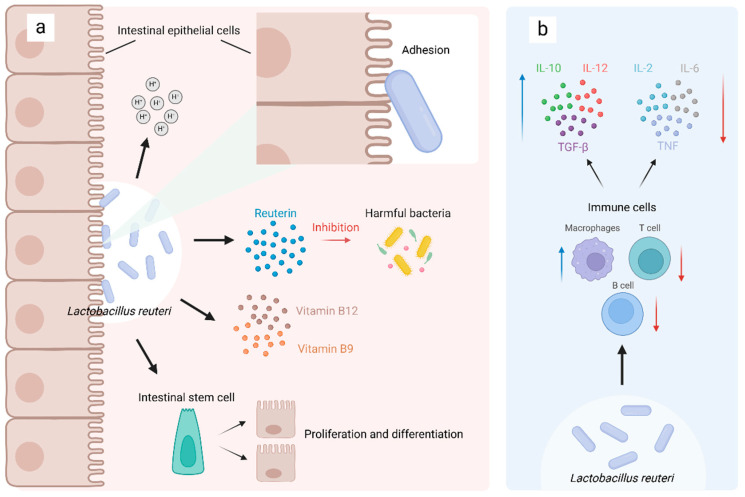
Mechanisms of action of *Lactobacillus reuteri*. The adhesion of *Lactobacillus reuteri* to the intestinal mucosa of humans and animals is a necessary prerequisite for its physiological functions. (**a**) In the gastrointestinal tract, the metabolites of *Lactobacillus reuteri*, such as reuterin, can inhibit the growth of harmful bacteria. Through the secretion of organic acids, it can reduce intestinal pH and promote the proliferation, differentiation, and maturation of intestinal epithelial cells, thereby enhancing the intestinal barrier. The synthesis of vitamins B9 and B12 by *Lactobacillus reuteri* is also beneficial to the organism. (**b**) *Lactobacillus reuteri* can also modulate the immune response. It can reduce the number of macrophages in the gastrointestinal tract, increase the numbers of B lymphocytes and CD4 T lymphocytes, and modulate immune cells to produce increased levels of anti-inflammatory mediators such as IL-10, IL-12, and TGF-β and decreased levels of pro-inflammatory mediators such as IL-2, IL-6, and TNF. Blue arrows indicate an increase in quantity, while red arrows indicate a decrease. Created in https://BioRender.com (accessed on 22 November 2024).

*Lactobacillus reuteri* can firmly adhere to the intestinal mucosa in both humans and animals. The adherence is multifaceted, involving non-specific physical interactions, such as hydrophobic forces, as well as the specific attachment mediated by cell wall components. This complex mechanism is essential for the bacterium to fulfil its physiological roles within the gastrointestinal tract [20,21,22]. Both hydrophobicity and adhesion are related to specific proteins on the cell surface, which are correlated [7,23,24,25,26,27]. Additionally, *Lactobacillus reuteri*, being a Gram-positive bacterium, may also contain pilins, which can promote adhesion [28] (Figure 2).

#### 2.2.1. Inhibiting the Growth of Harmful Bacteria

Reuterin, one of the main metabolites of *Lactobacillus reuteri*, can inhibit the growth of harmful bacteria such as *Escherichia coli*, *Clostridium perfringens*, and *Streptococcus pyogenes*, which is beneficial in maintaining the balance of the gastrointestinal microecology [29,30,31]. Reuterin is a class of metabolites produced in the process of glycerol fermentation by *Lactobacillus reuteri*, including 3-hydroxypropionaldehyde (3-HPA), 3-HPA dimer, 3-HPA hydrate, acrolein, and 3-hydroxypropionic acid. Among these metabolites, 3-HPA is one of the predominant antimicrobial components.

There are two main theories for the mechanism of the antimicrobial action of reuterin. One is that the electrophilic aldehyde group of reuterin can undergo a redox reaction with sulfhydryl-containing antioxidant molecules (such as glutathione and related enzymes), resulting in the entry of reactive oxygen species (ROS) into the cell, which leads to cell death [29,30,32]. The other is that the ribose-like structure of 3-HPA-dimer can specifically block ribonucleotide reductase from producing DNA, acting as a competitive inhibitor that disrupts DNA synthesis and gene expression, ultimately leading to cell death [29,33].

*Lactobacillus reuteri* also produces other potent antimicrobial compounds such as reutericin 6 and rotenocycline, which affect Gram-positive bacteria; phenyl lactic acid (PLA), which inhibits the growth of fungi and molds [34]; and the strong oxidant, hydrogen peroxide (H_2_O_2_), which is associated with human immune responses, ferroptosis, DNA damage, and other mechanisms [35,36].

#### 2.2.2. Strengthening the Intestinal Barrier

*Lactobacillus reuteri* enhances the intestinal barrier and repairs damaged intestinal mucosa effectively through complex mechanisms, which is of great significance for the function and health of the human digestive system. The mechanisms for enhancing the intestinal barrier include protecting the morphology of human intestinal organoids (hIOs) and ameliorating intestinal mucosal damage by promoting the proliferation and differentiation of intestinal epithelial cells [37,38], promoting the maturation of hIOs and intestinal epithelial cells by increasing the expression of mature intestinal-specific markers [39,40]. Ultimately, there is an increase in villus length, villus area, and the number and surface area of crypt-like outgrowth structures of hIOs. It also modulates pathological changes and proliferation levels, colon length, overall health, and body weight [41,42,43].

In addition, some organic acids produced by *Lactobacillus reuteri* fermentation, including lactic acid, acetic acid, propionic acid, and butyric acid, enhance the intestinal barrier through mechanisms such as lowering intestinal pH, stimulating digestive enzyme secretion, promoting intestinal growth and recovery, and maintaining the balance of the intestinal flora [44,45,46,47,48].

#### 2.2.3. Regulating the Immune Response

*Lactobacillus reuteri* can regulate immune responses and reduce inflammation by modulating both immune cells and cytokines. On the one hand, *Lactobacillus reuteri* can modulate the quantity and phenotype of immune cells. It reduces macrophages and increases B and CD4 T lymphocytes in the gastrointestinal tract [49,50]. In addition, it participates in macrophage phenotypic regulation by inhibiting M1-like macrophage markers and enhancing the expression of M2-like macrophage markers [50,51], and it promotes dendritic cell (DC) maturation through secreted metabolites or cell surface proteins [52,53]. On the other hand, it regulates the production of cytokines by immune cells, resulting in an increase in the production of anti-inflammatory mediators such as interleukin-10 (IL-10), IL-12, and transforming growth factor-beta (TGF-β), and a decrease in the production of pro-inflammatory mediators such as IL-2, IL-6, and tumor necrosis factor (TNF) [54,55,56]. The production of these cytokines results from a combination of the direct stimulation of immune cells and indirect stimulation of intestinal epithelial cells by *Lactobacillus reuteri*, which helps prevent chronic inflammation and tissue damage.

In addition, studies have shown that *Lactobacillus reuteri* strains can produce biogenic amines such as tyramine and histamine, which not only regulate inflammatory responses and influence various physiological processes as neurotransmitters, hormones, and neuromodulators, but also protect themselves from the acidic microenvironment formed during fermentation [57,58].

#### 2.2.4. Healthy Advantages

*Lactobacillus reuteri* has health benefits for humans in the synthesis of microorganisms, selenium metabolism, the lowering of lipids, blood sugar, and blood pressure, and fat reduction. *Lactobacillus reuteri* synthesizes vitamin B9 (folate) and vitamin B12 (cobalamin) and delivers these molecules to the small intestine through active metabolism, which is positive for promoting growth and development and maintaining health. The folate synthesized by *Lactobacillus reuteri* is monoglutamylated and polyglutamylated folate (also known as 5,10-toluene-THF or FPG), which is the simplest form of bioavailable folate. It is easily processed and absorbed by mammalian cells [59,60], which is important for the digestive, nervous, and reproductive systems [61,62].

*Lactobacillus reuteri* also accumulates and metabolizes selenium, converting inorganic selenium into organic selenium that can be utilized by the body. It inserts selenium into amino acids and forms selenomethionine (SeMet), selenocysteine (SeCys), and methylselenocysteine (MeSeCys) [63]. Organic selenium has higher bioavailability and higher activity, which has a positive effect on humans, animals, and plants [64,65,66,67]. Selenium is also important to *Lactobacillus reuteri*. Selenium can alter its tolerance to acid and bile and antibiotic resistance [68,69]. Moreover, it can enhance sugar and lipid metabolism in *Lactobacillus reuteri* [70,71] and induce a mild stress response [70].

*Lactobacillus reuteri* can reduce levels of lipids, primarily total cholesterol (TC) and low-density lipoprotein cholesterol (LDL-C), in the blood. The mechanisms may include the following: (1) The surface of the bacterial membrane can bind lipids (especially cholesterol). (2) Cholesterol is assimilated or converted to coprostanol by the probiotic cells, which reduces the absorption of cholesterol into the blood. (3) Short-chain fatty acids (SCFAs), bile acids (BA), branched-chain amino acids (BCAAs), propionic acid, and bile salt hydrolases (BSHs) produced by *Lactobacillus reuteri* have lipid-lowering effects [72,73,74,75,76].

*Lactobacillus reuteri* can regulate blood glucose, mainly by stimulating the intestinal secretion of glucagon-like peptide-1 (GLP-1) and GLP-2 and by increasing the secretion of insulin and C-peptide, which improve blood glucose control [77].

*Lactobacillus reuteri* can reduce blood pressure [78]. During fermentation, it releases active peptides with angiotensin-converting enzyme (ACE) inhibitory effects, known as ACE inhibitory peptides, which contribute to blood pressure reduction [79,80,81,82].

*Lactobacillus reuteri* can reduce body fat content and alleviate obesity because it improves digestive system function and reduces fat production. In addition, it also inhibits the expression of fatty-acid-production-related genes in certain tissues and reduces fat accumulation by upregulating AMP-activated protein kinase and acetyl-CoA carboxylase phosphorylation [83].

These above mechanisms are not independent of each other but interact together to some extent. For example, SCFAs, BA, and BCAAs have positive effects on the regulation of glucose and lipid metabolism. The regulation of blood glucose and blood lipids has a positive effect on fat reduction. The occurrence of hypertension, diabetes, cardiovascular disease, obesity, and other diseases is also the result of the interaction of these factors [84,85,86,87,88].

It should be emphasized that the physiological functions and mechanisms of action of *Lactobacillus reuteri* in different species (including humans, mice, pigs, chickens, sheep, cows, and horses) may vary [89,90,91]. For example, *Lactobacillus reuteri* regulates the balance of bacterial flora to improve growth performance and intestinal barriers in herbivores [92,93], while it regulates the function of immune response to improve health in humans [94,95]. In this article, we will mainly focus on the effects of *Lactobacillus reuteri* on human health.

## 3. Role of *Lactobacillus reuteri* in Oral Health

The oral microbiome is closely related to oral diseases. In the oral cavity of the human body, various microorganisms coexist in a balanced state in the normal state. However, when this balance is disrupted, certain microorganisms may lead to diseases such as gingivitis, periodontitis, cavities, and oral ulcers.

### 3.1. Lactobacillus reuteri and Periodontal Disease

Periodontal disease is one of the most common oral diseases in the human body, which can lead to chronic inflammation and tooth loss [96]. Due to the rapid increase in bacterial resistance to antimicrobial drugs, significant efforts have been made to reduce the use of antibiotics in the treatment of periodontal disease by shifting from treatment to prevention. Probiotics have been suggested as an alternative or supplement to traditional periodontitis treatments [97]. Research has extensively indicated that *Lactobacillus reuteri* is efficient as a therapeutic supplement and can be incorporated into the maintenance protocol following periodontal interventions. This probiotic not only reduces pathogenic bacteria in the oral cavity, but also contributes to the proliferation of beneficial microorganisms. *Lactobacillus reuteri* ATCC PTA 5289 can effectively inhibit various periodontopathic bacteria, including *Porphyromonas gingivalis* ATCC 33277, *Fusobacterium nucleatum* ATCC 25586, and *Prevotella intermedia* ATCC 25611 [98]. Some clinical research compared the *Lactobacillus reuteri* tablet experimental groups to the control groups and demonstrated that, despite no significant reduction in the gingival index (GI) and probing bleeding index (PI), there was a notable decrease in the levels of *Prevotella intermediate* in saliva and *Porphyromonas gingivalis* in subgingival samples [99].

The oral administration of *Lactobacillus reuteri* tablets is also effective as an adjuvant therapy for chronic periodontitis. It can continuously increase the number of beneficial bacteria in the mouth, helping to restore the natural microbial balance in the oral cavity [2]. In most patients with chronic periodontitis, it can significantly reduce the pro-inflammatory cytokine response (such as TNF-α, IL-1β, and IL-17) and improve the clinical indicators of periodontitis (including sulcus bleeding index SBI, periodontal probing depth PPD, clinical attachment level CAL), which may help to alleviate the activity of the disease [100,101,102,103,104,105].

Residual periodontal pockets are associated with an increased risk of periodontal disease progression and require additional treatment. *Lactobacillus reuteri* ATCC PTA 5289 and DSM 17938 can assist in the treatment of residual pockets with scaling and root planning (SRP), which can result in a greater reduction in pocket depth (PD) for moderate and deep pockets when compared to the placebo group [2].

In addition, some studies have shown that *Lactobacillus reuteri* also has a good clinical effect on peri-implantitis. Taking *Lactobacillus reuteri* buccal tablets in combination with oral mechanical debridement can improve the clinical parameters of implant mucositis or peri-implant mucositis. The effect lasts for at least 90 days, although it can only reduce the load of *Porphyromonas gingivalis*, and the microbiological effect is limited [106,107]. However, while *Lactobacillus reuteri* preparations have been shown to be a good choice for preventing the disease and improving the clinical parameters of healthy individuals [108], some studies also showed that the application of *Lactobacillus reuteri* ATCC PTA 5289 and DSM 17938 in the treatment of peri-implantitis did not have clinical and microbial benefits. In the study of Laleman et al., probiotics were directly dripped around the implant and then taken in the form of tablets [109] and, in the study of Peña et al., probiotic intervention was added on the basis of mechanical treatment and 0.12% chlorhexidine treatment [110]. However, both studies obtained negative results that *Lactobacillus reuteri* did not affect peri-implantitis.

Taken together, *Lactobacillus reuteri* has a positive effect on reducing oral harmful bacteria and improving clinical indicators of periodontal disease, and provides an auxiliary effect on the treatment of chronic periodontitis and peri-implantitis. Although its long-term efficacy needs to be further studied, the existing data support the potential application value of *Lactobacillus reuteri* as a supplement to traditional treatment.

### 3.2. Lactobacillus reuteri and Oral Mucosal Diseases

Oral mucosal diseases cover a wide range of common oral diseases affecting a wide range of people, including candidiasis, herpes simplex virus infection, recurrent aphthous ulcers, lichen planus, pemphigus vulgaris, and mucosal pemphigoid [111]. *Lactobacillus reuteri* has a promising potential in the treatment of these diseases. Studies showed that *Lactobacillus reuteri* DSM 17938 and ATCC PTA 5289 strains can protect the oral mucosa from 5-fluorouracil (5-FU) chemotherapy-induced injury by activating the cellular antioxidant defense system and by reducing the inflammatory response, and they have a protective effect on chemotherapy-induced oral mucosal injury [15].

Recurrent aphthous stomatitis (RAS) is a chronic inflammatory disease of the oral mucosa. It is characterized by painful oral ulcers that can occur in the general population [112]. The local probiotic nano-preparation extracted from *Lactobacillus reuteri* was used to treat recurrent stomatitis. Using probiotic nano-preparation for 7 consecutive days significantly reduced the size of the ulcer and the degree of pain. The effect was better than the traditional analgesic oral rinse [113]. However, the study of Pedersen et al. showed that, while probiotic tablets containing *Lactobacillus reuteri* could improve the ulcer severity score and reduce RAS-related oral pain within 3 months, the improvement effect was not statistically significant compared with the placebo group [114].

In vitro studies showed that *Lactobacillus reuteri* ATCC 55730 had an inhibitory effect on *Streptococcus mutans* and *Candida* [115]. *Lactobacillus reuteri* DSM 17938 and ATCC PTA 5289 showed an antifungal potential against five oral *Candida* species (*C. albicans*, *C. glabrata*, *C. tropicalis*, *C. dublin,* and *C. parapsilosis*) in vitro, and they almost completely inhibited the growth of *C. albicans* and *C. paraagglutinans* [116]. Consistently, in a randomized placebo-controlled clinical trial, oral *Candida albicans* load was significantly reduced in a group of frail elderly people after probiotic intervention using a composite lozenge containing *Lactobacillus reuteri* DSM 17938 and ATCC PTA 5289 [117].

Taken together, *Lactobacillus reuteri* has potential application value in treating oral mucosal diseases. To some extent, it can improve the clinical symptoms of recurrent stomatitis and other diseases by reducing the inflammatory response and protecting oral mucosa from damage, as well as inhibiting specific pathogens, although some of its effects still need further clinical verification.

### 3.3. Lactobacillus reuteri and Dental Caries

Dental caries is a multifactorial disease caused by the imbalance of the host oral flora, which is associated with increased levels of acidic and acid-resistant bacteria such as *Streptococcus mutans* and *Lactobacillus*. It is a major public health problem affecting 2.3 billion adults and 530 million children worldwide [118,119,120]. In response to this global problem, *Lactobacillus reuteri* has attracted the attention of researchers as a potential prevention and control strategy.

Studies showed that the oral supplementation of probiotic *Lactobacillus reuteri* ATCC 55730 in the first year after birth can reduce the incidence of dental caries in children at the age of 9 years [121]. Regular intake of *Lactobacillus reuteri* ATCC 55730 in adults can reduce the level of *Streptococcus mutans* in saliva [122]. Some in vitro studies have consistently shown that *Lactobacillus reuteri* strain ATCC PTA5289 has a significant anti-caries effect due to its ability to effectively interfere with the adhesion of *Streptococcus mutans* to saliva-coated hydroxyapatite [123]. In addition, the use of live *Lactobacillus reuteri* ATCC PTA5289 culture instead of its culture supernatant can significantly reduce the cariogenic activity of in vitro biofilms [124]. Moreover, the addition of the strain to the multi-species biofilm model also significantly reduced the number of *Streptococcus mutans* [125], further demonstrating its potential value in inhibiting plaque formation. Similarly, postbiotic mediators extracted from the supernatants of *Lactobacillus rhamnosus* and *Lactobacillus reuteri* could significantly inhibit the biofilm formation, metabolic activity, and expression of the key gene gtfB of *Streptococcus mutans*, suggesting that these postbiotic mediators may be used as an effective anti-biofilm agent to prevent dental caries [126].

Thus, *Lactobacillus reuteri*, as a potential strategy for the prevention and treatment of dental caries, has shown a certain effect by reducing the incidence of dental caries in children and adults, reducing the level of *Streptococcus mutans* in saliva, and inhibiting the formation and metabolic activity of biofilm. Although some research results have not reached statistical significance, the potential role of *Lactobacillus reuteri* in the prevention and management of dental caries is still worthy of further research and development.

### 3.4. Lactobacillus reuteri and Other Oral Diseases

In recent years, several studies have revealed the possible effect of *Lactobacillus reuteri* in improving other oral diseases. In a double-blind, randomized, and placebo-controlled trial, researchers investigated the effect of *Lactobacillus reuteri* supplements on oral wound recovery and postoperative discomfort after mandibular third molar extraction. The results showed that, although there were no significant differences between the probiotics group and the placebo group in terms of clinical wound healing rate, swelling degree, bacterial colonization, and salivary oxytocin level, the participants in the probiotics group reported significantly less swelling in the second week after the surgery, and the number of days of sleep disturbance and sick leave was also reduced. Thus, although *Lactobacillus reuteri* supplements had little effect on objective healing indicators, they may help to alleviate subjective discomfort after surgery, and this potential benefit deserves further study [127]. In addition, studies showed that the external use of *Lactobacillus reuteri* may be helpful for oral wound healing. Although the difference was not statistically significant compared with the placebo, the levels of some cytokines and chemokines in wound exudates in the probiotics group increased [128]. Therefore, larger-scale clinical trials are still needed to confirm the potential benefits of probiotics for oral wound healing.

Orthodontic treatment can readily cause the patient oral hygiene issues due to the difficulty of cleaning, which can result in flora imbalance. In a study of young people undergoing orthodontic treatment, researchers examined the effects of ingesting *Lactobacillus casei* or *Lactobacillus reuteri* lozenges for 14 consecutive days. The results showed that the periodontal statuses of the two treatment groups of patients were improved, and the effect of the *Lactobacillus reuteri* group was more significant, which could slightly but effectively improve the periodontal status of fixed orthodontic patients [129]. In addition, a study found that, in orthodontic patients using a mixture of *Lactobacillus reuteri*, three consecutive weeks of treatment significantly increased the pH of dental plaque. Despite the lack of a significant effect on the numbers of *Streptococcus mutans* and *Lactobacillus* in saliva and the microbial counts in dental biofilm in the short term, this study indicated that *Lactobacillus reuteri* had the potential to reduce the acidity of dental plaque [130]. In addition, a study showed that the short-term use of *Lactobacillus reuteri* Prodentis lozenges significantly improved oral hygiene and reduced the number of specific oral pathogens in patients undergoing fixed orthodontic treatment [131]. However, studies revealed that the use of *Lactobacillus reuteri* lozenges in patients with maxillary orthodontic appliances did not bring clinical and microbiological benefits [132].

Furthermore, taking *Lactobacillus reuteri* may also have an effect on improving bad breath [104,133]. A study showed that chewing gum containing *Lactobacillus reuteri* DSM 17938 and *Lactobacillus reuteri* ATCC PTA 5289 can reduce oral odor [134].

Overall, the application of *Lactobacillus reuteri* in oral health research is gradually expanding, and it has been proven to be beneficial for combating oral diseases such as periodontal disease, dental caries, and halitosis. Although most studies have a positive view of the effect of *Lactobacillus reuteri* in the treatment of oral diseases, it is worth noting that some studies have pointed out that *Lactobacillus reuteri* may not be able to exert the expected therapeutic effect, which may be related to the specificity of the patient’s microbial flora.

## 4. Role of *Lactobacillus reuteri* in Systemic Health

### 4.1. Lactobacillus reuteri and Gastrointestinal Diseases

*Lactobacillus reuteri*, a well-investigated probiotic strain, has demonstrated considerable efficacy in enhancing the equilibrium of intestinal microbiota and promoting gastrointestinal health. Studies have shown that *Lactobacillus reuteri* ATCC 55730 exhibits the capacity to diminish the colonization of *Helicobacter pylori* in the gastric environment and mitigate the symptoms of dyspepsia in vivo [18]. The rectal administration of *Lactobacillus reuteri* ATCC 55730 demonstrates efficacy in ameliorating inflammatory mucosa, reducing mucosal expression levels of inflammatory markers, and treating active distal ulcerative colitis in pediatric patients [14]. The probiotic strain *Lactobacillus reuteri* DSM 17938 has been found to modulate intestinal microflora composition in constipated children, stimulate gastrointestinal motility, and, subsequently, elevate defecation frequency [135]. Treatment with *Lactobacillus reuteri* LR92 DSM 26866 and DSM 17938 prevents abdominal colic in infants and reduces infant crying, severity of abdominal colic episodes in infants, and maternal postpartum depression [136,137,138]. *Lactobacillus reuteri* 4659, as a supplementary treatment, can significantly reduce the patient’s abdominal pain symptoms and reduce the level of inflammatory marker C-RP in the treatment of acute simple diverticulitis [139].

Overall, *Lactobacillus reuteri* has shown promising effects in the prevention and treatment of various gastrointestinal disorders, warranting further research to validate its efficacy and safety across different conditions.

### 4.2. Lactobacillus reuteri and Blood Glucose Regulation and Obesity

The potential of *Lactobacillus reuteri* in regulating blood glucose levels and ameliorating diabetes has garnered significant research interest. Notably, the SD5865 strain has exhibited the potential for elevating glucagon-like peptide-1 (GLP-1) and glucagon-like peptide-2 (GLP-2) levels, thereby stimulating insulin secretion [140]. Studies revealed that *Lactobacillus reuteri* GMNL-263 can protect diabetic rats from renal fibrosism [141], reduce blood glucose parameters, reverse the decline in liver antioxidant enzyme activity [142], and may prevent cardiomyocyte apoptosis [143]. While DSM 17938 does not affect HbA1c levels in patients with type 2 diabetes, it may improve insulin sensitivity [144]. The GL-104 strain has been shown to decrease fasting blood glucose levels in diabetic mice, improve glucose tolerance, and mitigate liver and kidney damage [145].

Furthermore, evidence suggests a correlation between intestinal flora and obesity, although the nature of this relationship may vary among individuals and studies [146]. Various strains of *Lactobacillus reuteri*, such as L3, J1, ADM14, SY523, HI120, and FGSZY33L6, can prevent weight gain, reduce fat mass, improve glucose homeostasis and insulin sensitivity, and regulate intestinal flora [77,83,147,148,149,150]. Some strains can also increase energy consumption, induce satiety hormone production, and inhibit food intake [149,151]. The probiotic function of *Lactobacillus reuteri* is not limited to weight management, but also reduces obesity-related complications such as depressive behavior and the inflammation of the liver and adipose tissue [152]. Nevertheless, not all *Lactobacillus reuteri* strains exhibit an effect on obesity. For instance, the L10 strain demonstrates no significant impact on obesity reduction [83].

### 4.3. Lactobacillus reuteri and Autism

Autism spectrum disorder (ASD) is a common neurodevelopmental disorder that may see improvements in symptoms by regulating the gut microbiome. In genetic, environmental, and idiopathic ASD mouse models, *Lactobacillus reuteri* treatment may help to address social deficits through the enhancement of social-interaction-induced synaptic activity in the ventral tegmental area via a vagus-nerve-dependent mechanism [153]. In addition, the strain may contribute to the restoration of the fear memory of the autism spectrum disorder model in Cntnap4 knockout male mice and could potentially improve behavioral abnormalities by regulating the balance of intestinal microflora [154]. Similarly, in the valproic acid-induced ASD-like model, the treatment with *Lactobacillus reuteri* and inulin for four consecutive weeks can ameliorate social defects, reduce brain inflammation, and help maintain the intestinal barrier [155].

Overall, *Lactobacillus reuteri* treatment appears to be a promising non-invasive microbial intervention that may help address autism-related social impairments, although further research is needed to confirm its efficacy.

### 4.4. Lactobacillus reuteri and Osteoporosis

Osteoporosis is a disease characterized by reduced bone mass and impaired bone strength, leading to an increased risk of fracture [156]. Studies have shown that *Lactobacillus reuteri* ATCC 6475 supplements can, to a certain extent, prevent bone loss caused by ovariectomy in mice. The strain reduced levels of the key markers of bone resorption, tartrate-resistant acid phosphatase 5 (TRAP5), and the receptor activator of nuclear factor-κB ligand (RANKL); reduced osteolysis; and inhibited the increase of CD4 T lymphocytes in bone marrow caused by castration [157]. In addition, the strain also increased trabecular bone mineral density in female mice with active inflammation [158]. Similarly, the findings from a clinical investigation indicated that the prolonged daily administration of this strain over a 12-month period in elderly female subjects can markedly attenuate the decrease in the total volumetric bone mineral density (vBMD) of the tibia [159]. Therefore, *Lactobacillus reuteri* may help to maintain bone health in elderly women and is expected to be a treatment for postmenopausal osteoporosis. However, more clinical data are needed to fully support its efficacy and safety.

### 4.5. Lactobacillus reuteri and Other Diseases

The efficacy of *Lactobacillus reuteri* treatment is not limited to the above areas, as it also shows a potential and positive impact on the treatment of various diseases and the regulation of immune response. Studies indicated that supplementation with *Lactobacillus reuteri* CRL1098 may improve the pathological conditions of mice and their offspring resulting from vitamin B12 deficiency [160]. *Lactobacillus reuteri* GMNL-263, to a certain extent, can reduce the levels of MCP-1, TNF, and IL-6 in the serum of mice fed with a high-fat diet and, concurrently, the biofilm formed by *Lactobacillus reuteri* 6475 can inhibit TNF generated by LPS-activated monocytes [161]. Furthermore, *Lactobacillus reuteri* may contribute to the enhancement of the pulmonary immune environment through the modulation of the gut microbiota, thereby mitigating the risk of asthma [162]. Moreover, engineered *Lactobacillus reuteri* WXD171-IsdB, as a vaccine delivery system, can effectively fight against *Staphylococcus aureus* infection and induce mucosal immune response [163], and, in a mouse model of acute liver injury, the culture supernatant of *Lactobacillus reuteri* ZJ617 can exert a protective effect [164].

**Figure 3:**
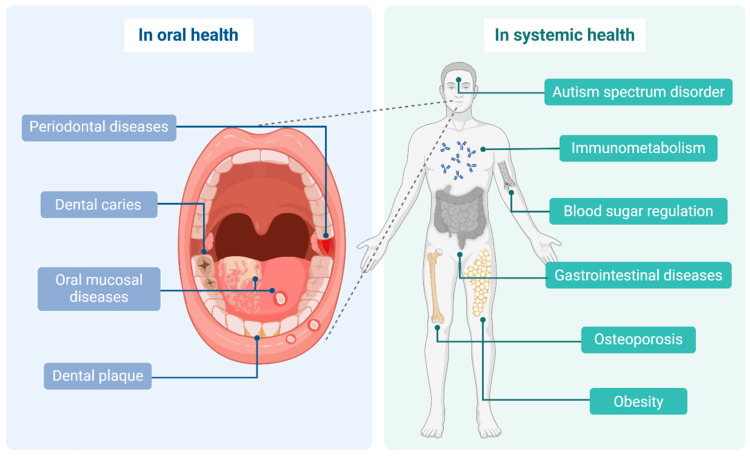
*Lactobacillus reuteri* affects both oral and systemic health. In oral health, it has an important impact on several diseases, including periodontal diseases, dental caries, dental plaque, and oral mucositis. In systemic health, it can affect gastrointestinal diseases, blood glucose regulation, obesity, autism spectrum disorder, osteoporosis, and immune response. Created in https://BioRender.com (accessed on 22 November 2024).

Overall, *Lactobacillus reuteri* appears to play an important role in maintaining systemic health. Its potential probiotic function is not limited to intestinal health and blood glucose regulation, but also extends to the protective effects of the respiratory tract, immune system, and liver. These multifaceted impacts provide a novel perspective and approach to the prevention and treatment of associated diseases (Figure 3).

## 5. Application and Prospect of *Lactobacillus reuteri*

### 5.1. Application of Lactobacillus reuteri in Food and Health Products

In recent years, nutritional and health products produced by probiotic fermentation have attracted much attention due to their direct and indirect advantages for human health, especially with *Lactobacillus reuteri*. Studies revealed that a variety of strains of *Lactobacillus reuteri* are safe for human consumption [165,166,167]. Based on the functions of *Lactobacillus reuteri*, such as improving microbial flora, enhancing the intestinal barrier, regulating immune response, and reducing inflammation, it is used in the production of fermented food. Its inclusion not only has a positive effect on gastrointestinal health and systemic health, but also prevents chronic diseases or improves the therapeutic effect. It can also improve the flavor and texture of food and prolong the storage time [168]. For example, during fermentation, the production of organic acids, ethanol, or antibacterial compounds can inhibit spoilage organisms and pathogens in fermented foods [169]; the essential amino acids, enzymes, and proteins can improve the nutritional value of foods [170].

Compared with natural products or products from artificial synthesis, *Lactobacillus reuteri*, as a kind of probiotic, has great advantages. For example, while folic acid produced by *Lactobacillus reuteri* does not mask the early hematological manifestations of vitamin B12 deficiency, vitamin intake through food may mask this manifestation. *Lactobacillus reuteri* can safely and effectively increase folic acid content during milk and soymilk fermentation [171,172,173,174]. In addition, selenium nanoparticles produced with lactic acid bacteria are less toxic than inorganic selenium forms, and they can alleviate cancer metastasis in vivo and in vitro [175]. Thus, using *Lactobacillus reuteri* and its metabolites as food additives or using genetic engineering technology to produce food or health products with *Lactobacillus reuteri* are promising approaches to improving human health [176].

### 5.2. Potential of Lactobacillus reuteri in Clinical Treatment of Oral Diseases

A large number of studies have shown that *Lactobacillus reuteri* exerts complex physiological functions through various mechanisms, which will have a significant effect on the treatment and prevention of many oral diseases. Whether it is used to develop new treatments or as an auxiliary method, it has great significance in maintaining oral health.

The application of *Lactobacillus reuteri* in the clinical treatment of oral diseases includes the following. (1) *Lactobacillus reuteri* can be used for the treatment and prevention of periodontal disease because of its ability to reduce the inflammatory response. Studies revealed that *Lactobacillus reuteri* has a significant effect in the treatment of chronic periodontitis as an adjuvant therapy for tooth washing and root planning, and can improve various clinical indicators related to periodontal health, including probing bleeding index, gingival index, bleeding on probing, periodontal probing depth, clinical attachment level, and gingival crevicular fluid volume [177,178,179,180]. For periodontal treatment, *Lactobacillus reuteri* can be made into tablets and sprays, and, for periodontal health care, it can be made into mouthwash and toothpaste. (2) *Lactobacillus reuteri* can reduce harmful bacteria in the oral cavity and supplement beneficial bacteria, which is expected to be used to treat or prevent dental caries. In recent years, people have become more interested in alternative methods aimed at restoring the balance of the oral microbiome. By using probiotics, the main pathogenic microorganism (*Streptococcus mutans*) is replaced from the dental niche to fight against oral diseases. Compared with the resistance of subgingival flora caused by systemic antimicrobial therapy, the use of *Lactobacillus reuteri* to treat oral diseases is more conducive to regulating the oral microecological balance. *Lactobacillus reuteri* has a significant effect in inhibiting *Streptococcus mutans* and *Streptococcus sobrinus* by effectively inhibiting the formation and adhesion of cariogenic biofilms, as shown in many studies [124,181,182,183]. (3) *Lactobacillus reuteri* can be used to treat oral mucosal diseases because of its function in regulating the immune system. On the one hand, *Lactobacillus reuteri* can directly play an immunoregulatory role in the oral cavity and reduce inflammation. Conversely, *Lactobacillus reuteri* can indirectly affect systemic health through the connection with the oral cavity and gastrointestinal tract, the “mouth-gut axis”, of which it is conducive to improving systemic health and fighting oral inflammation. It is believed that the application of *Lactobacillus reuteri* in the treatment of oral diseases can have a lasting impact on the oral flora after its stable colonization, which can be used as part of the post-intervention maintenance program [178].

### 5.3. Challenges and Future Directions of Lactobacillus reuteri Research

Studies have shown that probiotics play an important role in human health, especially oral health. Although *Lactobacillus reuteri* can reduce harmful bacteria in the mouth, such as *Porphyromonas gingivalis*, *Fusobacterium nucleatum*, and *Prevotella intermedia*, it also means changes in the oral flora. However, it is unknown whether and how this change causes the imbalance of oral flora. It is not known whether its acid-producing function causes caries. Currently, it is unclear whether the related products have functional consistency, stability, and safety, and how to control the application dose and frequency. These are some challenges researchers and clinicians face before putting *Lactobacillus reuteri* into clinical application. In addition, if *Lactobacillus reuteri* is applied to food fermentation, we need to also focus on the role of enzymes, as they will significantly affect the function, texture, taste, smell, and shelf life of fermented foods. Adding chemical or biological reagents and changing the living environment may be a good way to improve the functional activity of *Lactobacillus reuteri* and achieve better therapeutic or health effects.

## 6. Conclusions and Future Perspectives

In recent years, *Lactobacillus reuteri* has shown great potential in the research and application for oral health and even systemic health. As a probiotic, it can improve the oral flora, enhance the intestinal barrier, and regulate the immune response. These functions and mechanisms in the body are closely linked and play a key role in oral health problems like cavities, gum disease, mouth sores, and oral cancer. It even has an important impact on systemic health through mechanisms such as the mouth–gut axis. The impact is primarily shown in the synthesis of vitamins and metabolism of trace elements, as well as in the reduction in blood lipids, blood sugar, and blood pressure. Through these complex mechanisms, *Lactobacillus reuteri* can effectively prevent and treat oral diseases, especially dental caries and periodontal diseases, and related preparations have made some progress in clinical application. Oral care products containing *Lactobacillus reuteri*, such as lozenges, have been used to assist in the treatment of periodontal diseases and oral mucosal diseases.

In addition, the symbiotic properties and symbiosis among bacteria are important for *Lactobacillus reuteri* to fulfil its probiotic function. Using these properties, it is expected that more efficient and stable *Lactobacillus reuteri* products can be developed in the future. For example, symbiotic *Lactobacillus reuteri* NK33 and *Bifidobacterium adolescentis* NK98 can synergistically alleviate the occurrence of anxiety and colitis by modulating the gut immune response and microbial composition [184]. Mangiferin has shown excellent therapeutic value in the treatment of type-2-diabetic rats by promoting the proliferation of *Lactobacillus reuteri* 1-12 [185,186]. In addition, the symbiosis combination with inulin may also alleviate social barriers associated with ASD through the gut–brain axis [186].

Studies have also shown that changes in PH, temperature, oxidative stress, and ionic strength have a significant effect on the viability and activity of *Lactobacillus reuteri*, and, by using ultrasound and microencapsulation technology, the antioxidant capacity, acid tolerance, and resilience of *Lactobacillus reuteri* can be enhanced to increase the viability and bioactivity of *Lactobacillus reuteri*. By using dual-encapsulation technology and genetic engineering to modify *Lactobacillus reuteri*, metabolically active *Lactobacillus reuteri* can be targeted and delivered to oral lesions as an adjunctive therapy for oral diseases. These technologies are expected to provide more possibilities for the use of *Lactobacillus reuteri* in oral health [187,188].

However, the long-term safety of *Lactobacillus reuteri* remains to be investigated, especially its effect on oral flora structure and other probiotics. In order to effectively apply *Lactobacillus reuteri* to the clinical treatment of oral diseases, or as a self-management prevention strategy, more large-scale, long-term, randomized, and placebo-controlled clinical trials must be conducted in the future to determine the most effective strain combination, optimal carrier, and appropriate dose and frequency of administration of *Lactobacillus reuteri*. At the same time, attention should also be paid to the compliance and acceptance of *Lactobacillus reuteri* in patients of different age groups, as well as an in-depth understanding of its mechanism of action and host response, so as to tailor the optimal *Lactobacillus reuteri* treatment plan for different populations or individuals.

Furthermore, future research should also focus on assessing the biosafety of *Lactobacillus reuteri* and use multi-omics techniques such as genomics, proteomics, and metabolomics to deeply explore its mechanism of action. In addition, systematic clinical trials need to be carried out to verify its long-term safety and therapeutic effect, which is also the core of promoting the wide application of *Lactobacillus reuteri* in the prevention and treatment of oral diseases. Through these studies, more scientific and effective solutions for oral health and even systemic health are expected to be provided.

## Data Availability

Data sharing is not applicable to this article as no new data were created or analyzed in this study.
